# Decreased T2-signal intensities indicate positive response to front-line radiotherapy in pediatric low-grade gliomas

**DOI:** 10.1007/s11547-025-02118-4

**Published:** 2025-11-17

**Authors:** Simon Weiner, Monika Warmuth-Metz, Daniela Kandels, Beate Timmermann, Rolf-Dieter Kortmann, Stefan Dietzsch, Torsten Pietsch, Brigitte Bison, Mirko Pham, Astrid Katharina Gnekow, Annika Quenzer

**Affiliations:** 1 Department of Neuroradiology, University Hospital Würzburg, Josef-Schneider-Strasse 11, 97080 Würzburg, Germany; 2 Neuroradiological Reference Center for the Pediatric Brain Tumor (HIT) Studies of the German Society of Pediatric Oncology and Hematology, University Hospital Würzburg (Until 2020), Würzburg, Germany; 3 Swabian Children’s Cancer Center, Faculty of Medicine, University Augsburg, Augsburg, Germany; 4 Department of Particle Therapy, University Hospital Essen, Essen, Germany; 5 West German Proton Therapy Centre Essen (WPE), West German Cancer Centre (WTZ), German Cancer Consortium (DKTK) Center Essen, University Hospital Essen, Essen, Germany; 6 Department of Radiation Oncology, University Leipzig, Leipzig, Germany; 7 Institute of Neuropathology, DGNN Brain Tumor Reference Center, University of Bonn Medical Center, Bonn, Germany; 8 Department of Diagnostic and Interventional Neuroradiology, Faculty of Medicine, University Augsburg, Augsburg, Germany; 9 Neuroradiological Reference Center for the Pediatric Brain Tumor (HIT) Studies of the German Society of Pediatric Oncology and Hematology, University Augsburg, Faculty of Medicine (Since 2021), Augsburg, Germany

**Keywords:** Pediatric low-grade glioma, Magnetic resonance imaging, Radiotherapy, Response assessment, T2-weighted imaging

## Abstract

**Purpose:**

To evaluate MRI changes in T2-weighted imaging (T2WI) signal intensity (T2SI) as a potential imaging marker for assessing response to radiotherapy (RT) in pediatric low-grade glioma (pLGG).

**Materials and methods:**

This retrospective study analyzed imaging data of 56 pLGG patients (mean age, 12.4 ± 3.5 years; 33/56 [58.9%] male) treated with photon-based or proton-based RT within the SIOP-LGG 2004 study and registry. Tumor signal characteristics on T2WI were qualitatively and quantitatively assessed at baseline and up to 24 months post-RT. Tumor volumes were calculated, and correlations between ∆T2SI and volumetric changes were examined. Statistical tests included inferential tests, correlation analysis, and linear regression.

**Results:**

At baseline, 87.5% tumors were rated as hyperintense, while none was rated hypointense. The mean ratio between T2SI of the tumors compared to the cerebral cortex was 1.70. A significant decrease in T2SI was observed over time with the strongest decrease at 24 months post-RT (− 18.7%; *p* = 0.002). ∆T2SI correlated significantly with tumor volume reduction (r = 0.46, *p* < 0.001) and response assessment (ρ = 0.51, *p* < 0.001). There was no significant influence of age, sex, tumor location, histology, or RT type on ∆T2SI. Cases of pseudoprogression cases exhibited stable T2SI despite transient increases in contrast enhancement or tumor volume.

**Conclusion:**

A reduction in T2SI was consistently associated with tumor volume reduction, suggesting that a decrease in T2SI may serve as an additional imaging marker of a positive response to RT in pLGG patients.

## Introduction

Pediatric low-grade glioma (pLGG) are the most common brain tumors in childhood and adolescence, with pilocytic astrocytoma being the largest subgroup [1]. While the majority of tumors may be surgically resected, non-surgical treatment modalities are offered for symptomatic and/or progressive, non-resectable pLGG in current therapy algorithms [2–4]. Radiotherapy (RT) has traditionally been employed as front-line treatment, while its contemporary indications are considerably restricted and chemotherapy and/or targeted treatment are preferred to reduce long-term neuro-cognitive deficits [5–7]. Following non-surgical treatment, the evolution of clinical symptoms and radiologic tumor response may not always be concordant [8]. Thus, evaluation of post-treatment MR images is crucial for response assessment.

On MRI, pLGG are characterized by low signal intensities on T1-weighted images (T1WI) and high signal intensities on T2-weighted images (T2WI) [9, 10]; the apparent diffusion coefficient is higher compared to high-grade glioma [11]. In contrast to diffuse LGG, pilocytic astrocytomas are well-demarcated and predominantly strongly contrast-enhancing tumors [9, 12]. However, contrast enhancement may change spontaneously during follow-up without any prognostic effect [13]. Therefore, the decrease or increase of contrast-enhancement in pLGG should not be used as surrogate marker for response to therapy or progression, as they are in high-grade glioma. The recently published guideline of the international pediatric neuro-oncology (RAPNO) working group recommended rather measuring the tumor on T2WI to assess size changes in pLGG [14].

Especially following RT, imaging for response assessment in pLGG has to consider interfering phenomena like pseudoprogression with increasing contrast-enhancement, tumor volume, and perifocal tumor edema being characteristic features [15–18]. Recent studies have evaluated T2WI alongside post-contrast images [15–17]. Yet, in contrast to the well-documented challenges in differentiating post-radiation effects from true progression, the literature provides limited insight into tumor response patterns, particularly regarding concomitant changes in T2-signal intensity (T2SI) following therapy. This gap underscores the need for additional response markers that are accessible, easily interpretable, and reliable in clinical practice. Given the relevance of changing tumor volume on T2WI for response assessment in pLGG, we aimed to investigate post-treatment changes of T2SI qualitatively and quantitatively in a previously identified cohort after front-line RT within the SIOP-LGG 2004 trial and LGG-registry of the German Society of Pediatric Hematology and Oncology. We hypothesized that T2SI reduction would correspond to the extent of tumor volume reduction and could potentially serve as a complementary radiological biomarker in treatment monitoring.

## Materials and methods

### Patients

For this retrospective analysis, data and imaging material were evaluated from LGG patients diagnosed under the age of 18 years and treated with front-line photon- (XRT) or proton-based RT (PBT) within the prospective multicenter SIOP-LGG 2004-study (registration from 4/2004 to 03/2012, ClinicalTrials.gov PRS NCT00276640, EudraCT 2005-005377-29 [19]), and the subsequent LGG-registry (recruiting from 4/2012 to 12/2018). The histologic diagnosis of LGG was recorded based on central review and classified according to the WHO classification valid at the time of diagnosis. In defined cases, diagnosis based on imaging criteria was accepted when no histopathological sampling was performed. Informed consent had been obtained from each patient or legal guardian when entering the study or registry. Neuroimaging data were obtained from the Neuroradiological Reference Center database. Data from the irradiated LGG-study and registry patient cohort, including details on radiation techniques, have been published previously [15]. For this analysis, we included intracranial pLGG, including optic pathway gliomas, with clinically and radiologically defined positive responses [14, 27]. Patients treated with interstitial radiotherapy (125-iodine-seeds), with concomitant chemotherapy, progressive disease after RT and non-determinable response to therapy after 24 months post-RT were excluded.

### Imaging analysis

One board-certified neuroradiologist (A.Q.) and one fourth year resident (S.W.) performed the MR-imaging analysis. Furthermore, a senior neuroradiologist (M.W.) rated debatable cases. Tumor localization was assessed as brainstem, cerebellum, cerebral hemisphere, supratentorial midline (SML) outside the optic pathways or SML within the optic pathways (including hypothalamus). MRI data were evaluated pre-RT (baseline; time-point t0); at first follow-up within the first six months post-RT (t6); 12 months post-RT (t12), 18 months post-RT (t18) and 24 months post-RT (t24). Evaluation included tumor volume calculation using a commonly applied approximation formula (a × b × c × 0.5). Radiological response was assessed by comparing the tumor volume in the last follow-up MRI to the baseline scan, with response categories adapted from the RAPNO response criteria [14]: complete response (CR), partial response (PR; 50% or greater tumor reduction), minor response (MR; 25–49% tumor reduction), and stable disease (SD). SD was considered a positive response due to its clinical benefit, despite the absence of a significant reduction in tumor volume, in line with the commonly accepted practice in LGG-studies. Furthermore, the following imaging features were evaluated: The presence of calcification or hemorrhage was assessed on T1WI or SWI/T2*-sequence and categorized as intratumoral susceptibility. Visual (qualitative) assessment of T2SI as hyper-, iso- or hypointense in comparison to the supratentorial cortex on baseline imaging; and as decrease, increase or without significant change of signal intensity on follow-up compared to the baseline examination. The T2SI of the tumor was quantitatively determined for all baseline and follow-up MRI datasets. The raters placed circular, two-dimensional regions of interest (ROIs) within the tumor by referencing anatomical landmarks and the relative position within the tumor, as well as in the cerebral cortex, using the axial T2-weighted sequence. If additional T2-weighted sequences in other planes (coronal or sagittal) were available, they were used only in cases where the axial T2WI was unavailable and were consistently applied throughout follow-up. Tumor ROIs sampled solid, representative T2 signal—preferentially within enhancing tissue when present—while avoiding cystic/necrotic areas and susceptibility foci. Furthermore, to minimize variability in the measurements, the size of the ROIs was kept consistent throughout all scans whenever feasible. In cases where a decrease in tumor volume necessitated adjustments, ROI dimensions were modified minimally and only to the extent required to ensure placement within the solid tumor tissue and to avoid inclusion of surrounding non-tumoral areas. The mean value of the signal intensity from each ROI was extracted. The parameter T2SI was calculated based on these ROIs by the following formula:

$$T2SI = \frac{1}{2} \left( {\frac{{ROI_{tumor} \left( {{\text{rater }}1} \right)}}{{ROI_{cortex} \left( {{\text{rater }}1} \right)}} + \frac{{ROI_{tumor} \left( {{\text{rater }}2} \right)}}{{ROI_{cortex} \left( {{\text{rater }}2} \right)}}} \right)$$

For follow-up examinations, the change in T2SI relative to baseline was defined as ΔT2SI. ΔT2SI was calculated at each follow-up time-point by comparison with the baseline scan. The last available follow‑up time-point varied because not all MRIs within the 24 months post‑RT period were archived in the reference database. For correlation and regression analyses, both ΔT2SI and tumor volume change were extracted from the last available follow‑up within 24 months after RT (t6, t12, t18 or t24) for each patient. In patients with CR, we used the last MRI with visible residual tumor to evaluate ΔT2SI.

**Fig. 1:**
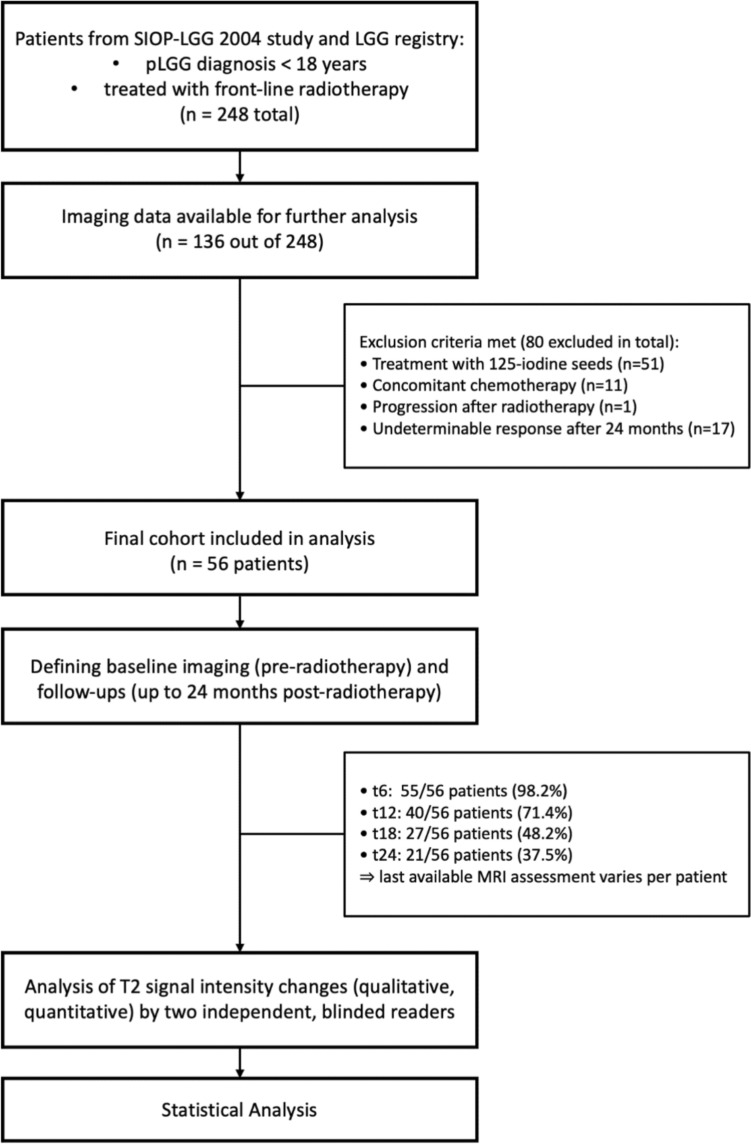
Flowchart of the study design

Pseudoprogression was defined according to the criteria by Stock et al. (increase of enhancement and/or T2-lesions after RT with spontaneous stabilization or improvement) [15]. Undeterminable cases were excluded. Parameters of scanners and T2WI, like magnetic field strength, time to echo and time to repetition, were documented for each examination. A summary of the study design is presented as a flowchart in Fig. 1.

### Statistics

Statistical analyses and visualizations were performed using R, version 4.4.2 (R Foundation for Statistical Computing, Vienna, Austria). All results were documented as n (%) or mean ± standard deviation unless otherwise specified. Testing for normal distribution was performed via QQ-plots for all continuous variables. 95% confidence intervals (95%-CI) were documented if appropriate. Probability values of *p* < 0.05 were considered significant. The inter-rater reliability of quantitative measurements was estimated via inter-rater intra-class correlation (ICC; two-way, agreement) and interpreted according to Koo and Li 2016 [20]. A receiver operating characteristic (ROC) analysis with the area under the curve (AUC) was calculated to assess the agreement between qualitative and quantitative ratings. Wilcoxon-signed-rank-test, Kruskal–Wallis-test or Mann–Whitney-U-test were used to compare differences between independent or dependent samples, respectively. Pearson’s correlation coefficient r or Spearman’s correlation coefficient ρ was calculated for the following variable pairs: r(ΔT2SI, relative tumor volume change), r(baseline T2SI, ΔT2SI); r(baseline T2SI, relative tumor volume change), and ρ(ΔT2SI, radiological response assessment). Correlations with field strength, echo time, and repetition time were performed to assess potential dependencies of T2SI on acquisition parameters in this multicenter dataset: ρ(T2SI, magnetic field strength), r(T2SI, echo time), r(T2SI, repetition time). A simple linear regression model was used to investigate the predictive value of ΔT2SI on tumor volume reduction, with tumor volume change as the dependent variable and ΔT2SI as the independent variable. The German LGG studies did not include predefined radiological questions. Therefore, all analyses were exploratory, and *p*-values were considered descriptive measures to detect and study meaningful effects.

## Results

### Study cohort

Imaging data meeting the inclusion criteria of our previous analysis had been available for 136/248 patients with front-line RT from the SIOP-LGG 2004-study and LGG-registry [11]. 80 of these 136 patients were excluded for treatment with 125 iodine seeds (n = 51), concomitant chemotherapy (n = 11), progression (n = 1) or undetermined response assessment (n = 17). Thus, MRI datasets of 56 pLGG patients treated with conventional dose RT (XRT: n = 39 [69.6%], PBT: n = 17 [30.4%]) were analyzed. Epidemiologic data of the study cohort and tumor characteristics are detailed in Table 1. Of the 56 patients, 55 (98.2%) had a follow-up MRI at t6; 40 (71.4%) at t12; 27 (48.2%) at t18 and 21 (37.5%) at t24. Thirteen of the 56 (23.2%) patients had four, 17 patients (30.4%) three, 14 patients (25.0%) two and 12 patients (21.4%) had only one follow-up MRI. As not all MRIs within the first 24 months post-RT were available in the Neuroradiological Reference Center database, the latest assessment time-point varied between patients (t6 for n = 11 [19.6%], t12 for n = 11 [19.6%], t18 for n = 13 [23.2%], t24 for n = 21 [37.5%]).

**Table 1:** Overview of the study cohort and tumor characteristics

Parameter	Mean ± SD or n (%)
Age at diagnosis [years]	10.7 ± 3.6 (range: 3–17)
Age at start of first line radiation therapy [years]	12.4 ± 3.5 (range: 4–21)
*Sex*	
Female	23 (41.1)
Male	33 (58.9)
*Tumor localization*	
Brainstem	17 (30.4)
Cerebellum	5 (8.9)
Cerebral hemispheres	5 (8.9)
Supratentorial midline, outside the optic pathways	4 (7.1)
Supratentorial midline, optic pathways	24 (42.9)
Dodge I	8 (14.3)
Dodge II	2 (3.6)
Dodge III	14 (25.0)
Other *	1 (1.8)
*Tumor histology*	
Pilocytic astrocytoma WHO grade I	34 (60.7)
Pilomyxoid astrocytoma WHO grade I	2 (3.6)
Astrocytoma not otherwise specified	3 (5.4)
Pleomorphic xanthoastrocytoma WHO grade II	2 (3.6)
Ganglioglioma	4 (7.1)
Rosette-forming glioneuronal tumor WHO grade I	1 (1.8)
Radiological diagnosis	10 (17.9)
No histological examination performed	9 (16.1)
No detection of tumor cells in histological analysis	1 (1.8)
Tumor volume at baseline imaging [cm^3^]	7.0 ± 11.2 (range: 0.1–67.8)
*Type of radiotherapy*	
Photon-based radiotherapy (median dose 54.0 Gy; range 46.8–59.4 Gy)	39 (69.6)
Proton-based radiotherapy (median dose 54.0 Gy; range 50.4–54.0 Gy)	17 (30.4)
*True pseudoprogression* (Stock et al. [15])	
Yes	25 (44.6)
No	31 (55.4)
*Response assessment adapted to RAPNO-criteria* (Fangusaro et al. [14])	
Stable disease	16 (28.6)
Minor response	13 (23.2)
Partial response	26 (46.4)
Complete response	1 (1.8)
*Response with respect to tumor volume assessment*	
Reduction of tumor volume (minor or partial or complete response)	40 (71.4)
Stabilization of tumor volume (stable disease)	16 (28.6)

^*^The primary tumor was described as located in the fourth ventricle, but the MRI at the time of diagnosis was not available in our database to confirm or specify the exact localization. The residual tumor that was irradiated was located along the dorsal brainstem

### Analysis of T2SI in baseline imaging

**Fig. 2:**
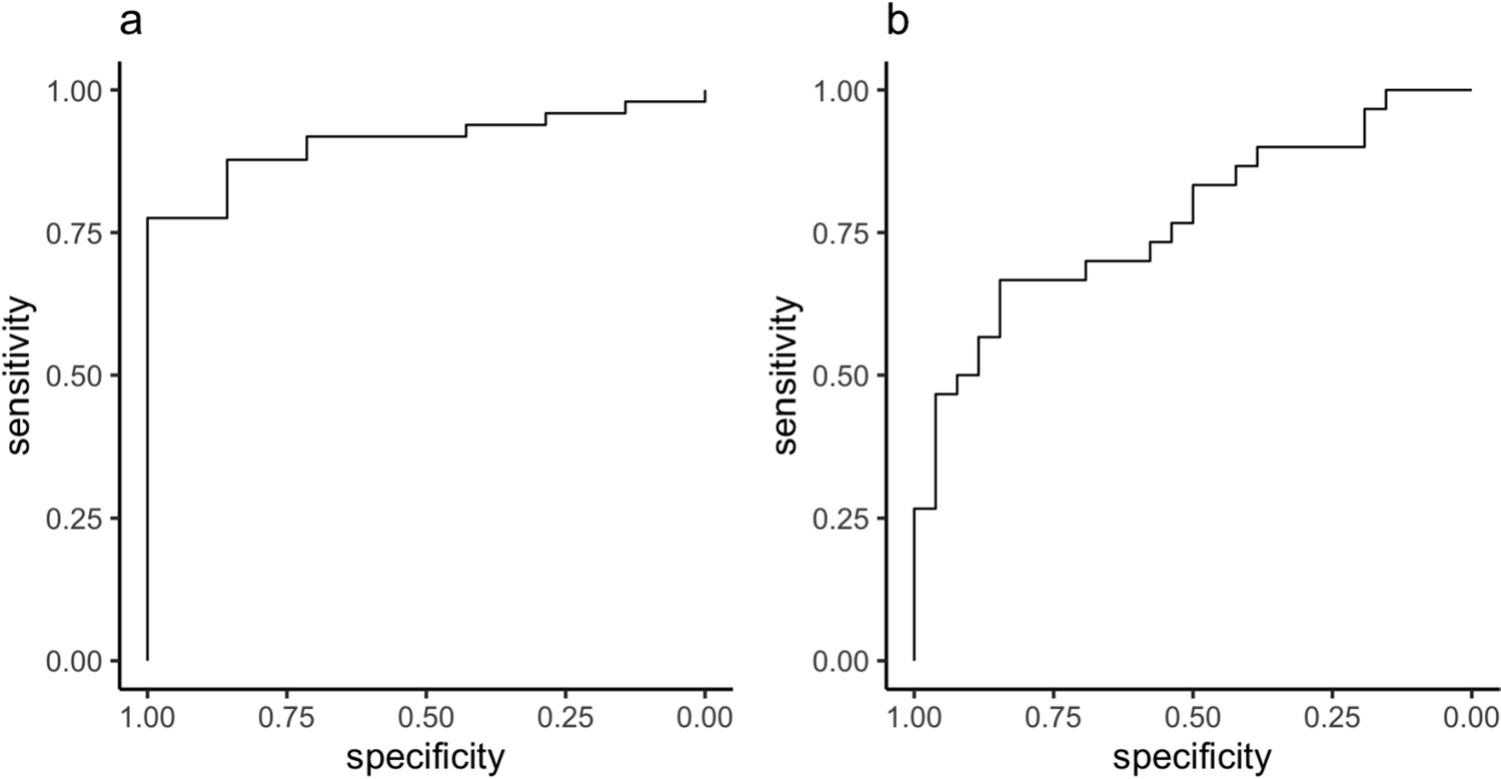
Receiver operating characteristic (ROC) curves illustrating the agreement between qualitative and quantitative assessments of T2 signal intensity (SI). **a** ROC curve for baseline imaging: Predicting visually classified hyperintensity from quantitative SI measurements achieved an area under the curve (AUC) of 0.91 (95% CI: 0.83–0.99), indicating good to excellent agreement. **b** ROC curve for follow-up imaging: Predicting visually classified T2SI reduction from quantitative ΔSI measurements yielded an AUC of 0.77 (95% CI: 0.65–0.89), indicating moderate to good agreement

Qualitative analysis of the baseline MRI datasets classified 7/56 (12.5%) as isointense and 49/56 pLGG (87.5%) as hyperintense in comparison to the cerebral cortex; no pLGG appeared hypointense. At baseline, mean T2SI was 1.70 ± 0.44 (95%-CI: 1.59, 1.82) for all 56 patients. The inter-rater ICC for quantitative T2SI measurements was 0.73. An agreement analysis between the qualitative and quantitative assessments, conducted via ROC analysis (Fig. 2a), indicates that predicting visually classified hyperintensity based on quantitative T2SI measurements yields an AUC of 0.91 (95%-CI: 0.83, 0.99). There was no significant correlation between T2SI and the scanning parameters magnetic field strength (ρ =  − 0.01, *p* = 0.92), echo time (r =  − 0.01, *p* = 0.92) or repetition time (r =  − 0.04, *p* = 0.77). Tumors with higher T2SI at baseline exhibited a stronger decrease in T2SI following RT: r(T2SI, ΔT2SI) =  − 0.32, *p* = 0.015. However, baseline T2SI did not significantly correlate with post-RT tumor volume reduction (r =  − 0.19, *p* = 0.15).

### Analysis of T2SI in follow-up imaging

Qualitative assessment revealed a decrease of T2SI in 30/56 patients (53.6% and most cases achieved PR as shown in Table 2), no relevant changes in 25/56 patients (44.6%) and an increase in 1/56 patient (1.8%) on the latest available follow-up MRI. The tumor with increased T2SI was rated as having pseudoprogression at that time-point.

**Table 2:** Qualitative ratings of T2SI and **Δ**T2SI across subgroups

Sub-cohort	Qualitative rating of T2SI: n (%)			ΔT2SI	*p*
	↓	↔	↑	(mean ± SD)	
*Sex*					
Female (n = 23)	11 (47.8)	11 (47.8)	1 (4.3)	− 0.141 ± 0.217	0.37^ m^
Male (n = 33)	19 (57.6)	14 (42.4)	0	− 0.209 ± 0.237	
*`Tumor localization*					
Brainstem (n = 17)	10 (58.8)	6 (35.3)	1 (5.9)	− 0.213 ± 0.179	0.96^ k^
Cerebellum (n = 5)	4 (80.0)	1 (20.0)	0	− 0.165 ± 0.159	
Cerebral hemispheres (n = 5)	1 (20.0)	4 (80.0)	0	− 0.147 ± 0.149	
SML, outside the optic pathways (n = 4)	2 (50.0)	2 (50.0)	0	− 0.211 ± 0.254	
SML, optic pathways (n = 24)	12 (50.0)	12 (50.0)	0	− 0.156 ± 0.289	
Other* (n = 1)	1 (100.0)	0	0	− 0.353	
*Tumor histology*					
PA WHO grade I (n = 34)	21 (61.8)	13.(38.2)	0	− 0.185 ± 0.227	0.52^ k^
PMA WHO grade I (n = 2)	1 (50.0)	1 (50.0)	0	− 0.064 ± 0.059	
Astrocytoma nos (n = 3)	1 (33.3)	2 (67.7)	0	− 0.286 ± 0.269	
PXA WHO grade II (n = 2)	0	2 (100.0)	0	− 0.224 ± 0.146	
Ganglioglioma WHO grade I (n = 4)	2 (50.0)	1 (25.0)	0	− 0.089 ± 0.206	
RGNT WHO grade I (n = 1)	0	1 (100.0)	1 (25.0)	− 0.392	
No histology (n = 10)	5 (50.0)	5 (50.0)	0	− 0.166 ± 0.289	
*Intratumoral susceptibility at baseline*					
Yes (n = 15)	8 (53.3)	7 (46.7)	0	− 0.200 ± 0.225	0.39^ m^
No (n = 41)	22 (53.7)	18 (43.9)	1 (2.4)	− 0.174 ± 0.233	
*Type of radiotherapy*					
Photon-based (n = 39)	22 (56.4)	17 (43.6)	0	− 0.191 ± 0.254	0.42^ m^
Proton-based (n = 17)	8 (47.1)	8 (47.1)	1 (5.9)	− 0.157 ± 0.161	
*True pseudoprogression*					
Yes (n = 25)	15 (60.0)	9 (36.0)	1 (4.0)	0.191 ± 0.242	0.91^ m^
No (n = 31)	15 (48.4)	16 (51.6)	0	− 0.172 ± 0.222	
*Response assessment*					
SD (n = 16)	1 (6.3)	14 (87.5)	1 (6.3)	− 0.021 ± 0.187	**0.002**^** k**^
MR (n = 13)	8 (61.5)	5 (38.5)	0	− 0.153 ± 0.210	
PR (n = 26)	20 (76.9)	6 (23.1)	0	− 0.283 ± 0.207	
CR (n = 1)	1 (100.0)	0	0	− 0.444	
*Response with respect to tumor volume assessment*					
Stabilization (SD; n = 16)	1 (6.3)	14 (87.5)	1 (6.3)	− 0.021 ± 0.187	** < 0.001**^** m**^
Reduction (MR/PR/CR; n = 40)	29 (72.5)	11 (27.5)		− 0.245 ± 0.214	

Results are based on the last available MRI for each of the 56 cases, relative to baseline. Values are reported as n (%) or mean ± SD, as appropriate

^k^: *p*-value is calculated via Kruskal–Wallis-test; ^m^: *p*-value is calculated via Mann–Whitney-U-test; *p*‑values refer to quantitative ΔT2SI; visual ratings are descriptive only; ↓: decrease; ↔ : no significant change; ↑: increase. * unknown exact localization as described in Table 1

*T2SI* Signal intensity of the tumor in the last available T2 weighted imaging compared to baseline; *ΔT2SI* Difference of the signal intensity of the tumor in the last available dataset compared to baseline; *SML* supratentorial midline; *PA* Pilocytic astrocytoma; *PMA* Pilomyxoid astrocytoma; *nos* Not otherwise specified; *PXA* Pleomorphic xanthoastrocytoma; *RGNT* Rosette-forming glioneuronal tumor; *SD* Stable disease; *MR* Minor response; *PR* Partial response; *CR* Complete response

ΔT2SI of each tumor showed a significant decrease of mean ± SD − 0.181 ± 0.229 (*p* < 0.001) compared to the corresponding baseline MRI. Tumor volume reduction did not differ significantly between isointense and hyperintense tumors in baseline imaging (*p* = 0.15). A minimal reduction in the quantitative assessment was even observed in the single tumor that was qualitatively rated as having increased T2SI (ΔT2SI: − 0.007). The inter-rater ICC for ΔT2SI measurements was 0.76 for all follow-up measurements and 0.80 for the last available follow-up dataset. An agreement analysis between the qualitative and quantitative follow-up assessments (Fig. 2b) indicated that predicting a visually classified decrease in T2SI from quantitative measurements achieved an AUC of 0.77 (95%-CI: 0.65, 0.89). T2SI did not change significantly at t6 (ΔT2SI_t6_: mean ± SD − 0.033 ± 0.222; 95%-CI − 0.093, 0.027; *p* = 0.17). T2SI significantly decreased at t12 (ΔT2SI_t12_: mean ± SD − 0.104 ± 0.271; 95%-CI − 0.190 − 0.017; *p* = 0.009), t18 (ΔT2SI_t18_: mean ± SD − 0.184 ± 0.243; 95%-CI − 0.280, − 0.088; *p* < 0.001) and t24 (ΔT2SI_t24_: mean ± SD − 0.187 ± 0.246; 95%-CI − 0.299, − 0.075; *p* = 0.002) compared to baseline, respectively.

**Fig. 3:**
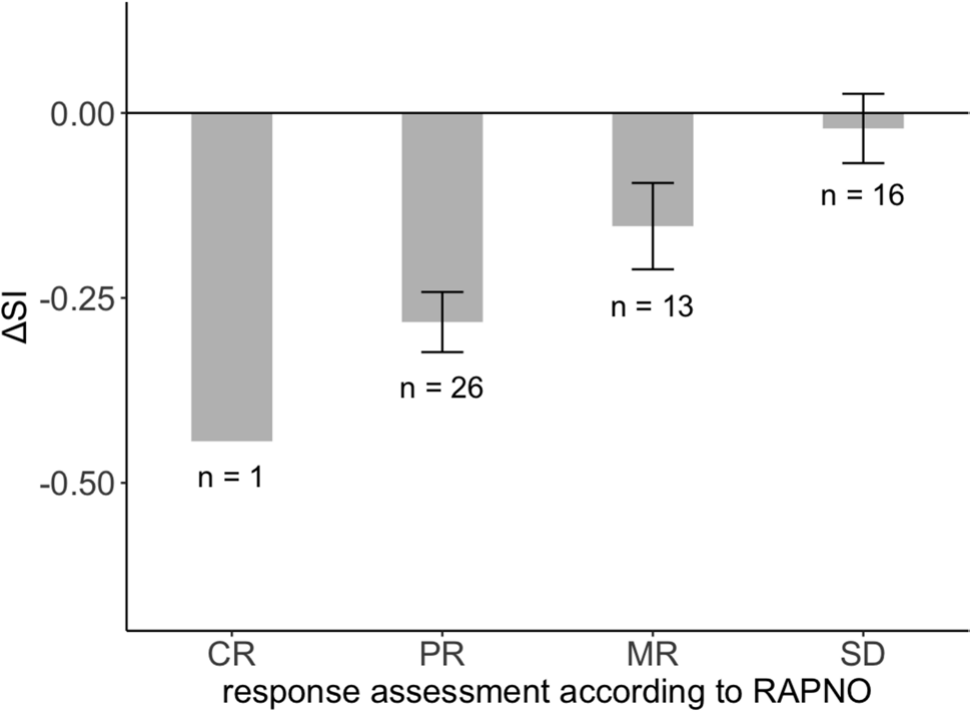
The parameter ΔT2SI shows significant correlation with response assessment subgroups (ρ = 0.51; *p* < 0.001). The grey bars represent the mean, the smaller black bars represent the standard error of the mean for each value. *ΔSI* Difference of tumor signal intensities in T2-weighted imaging in the last available MRI compared to baseline MRI pre-radiotherapy; *SD* Stable disease; *MR* Minor response; *PR* Partial response; *CR* Complete response

**Fig. 4:**
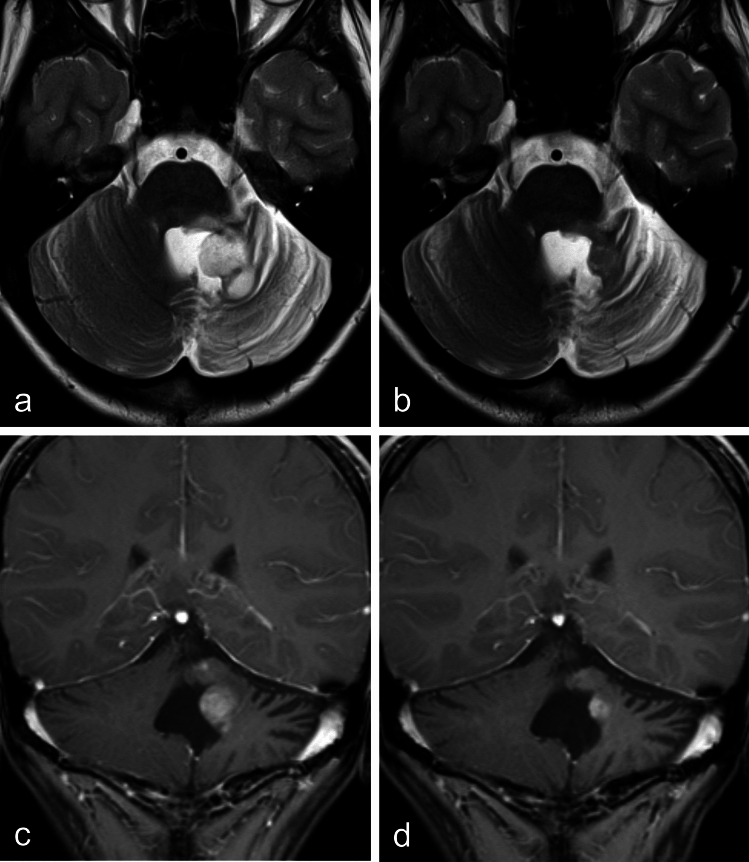
Case of partial response. A 7-year-old girl was diagnosed with a pilocytic astrocytoma. **a** Following incomplete resection, local progression was observed four years later. The residual tumor appeared hyperintense on T2-weighted images compared to the parenchyma of the unaffected right cerebellar hemisphere. Conventional photon radiotherapy was then initiated. **b** Two years after radiotherapy, both the tumor size and the T2 signal intensity had significantly decreased. **c** Contrast-enhancing tumor portions on baseline MRI were still present two years post-therapy (**d**)

**Fig. 5:**
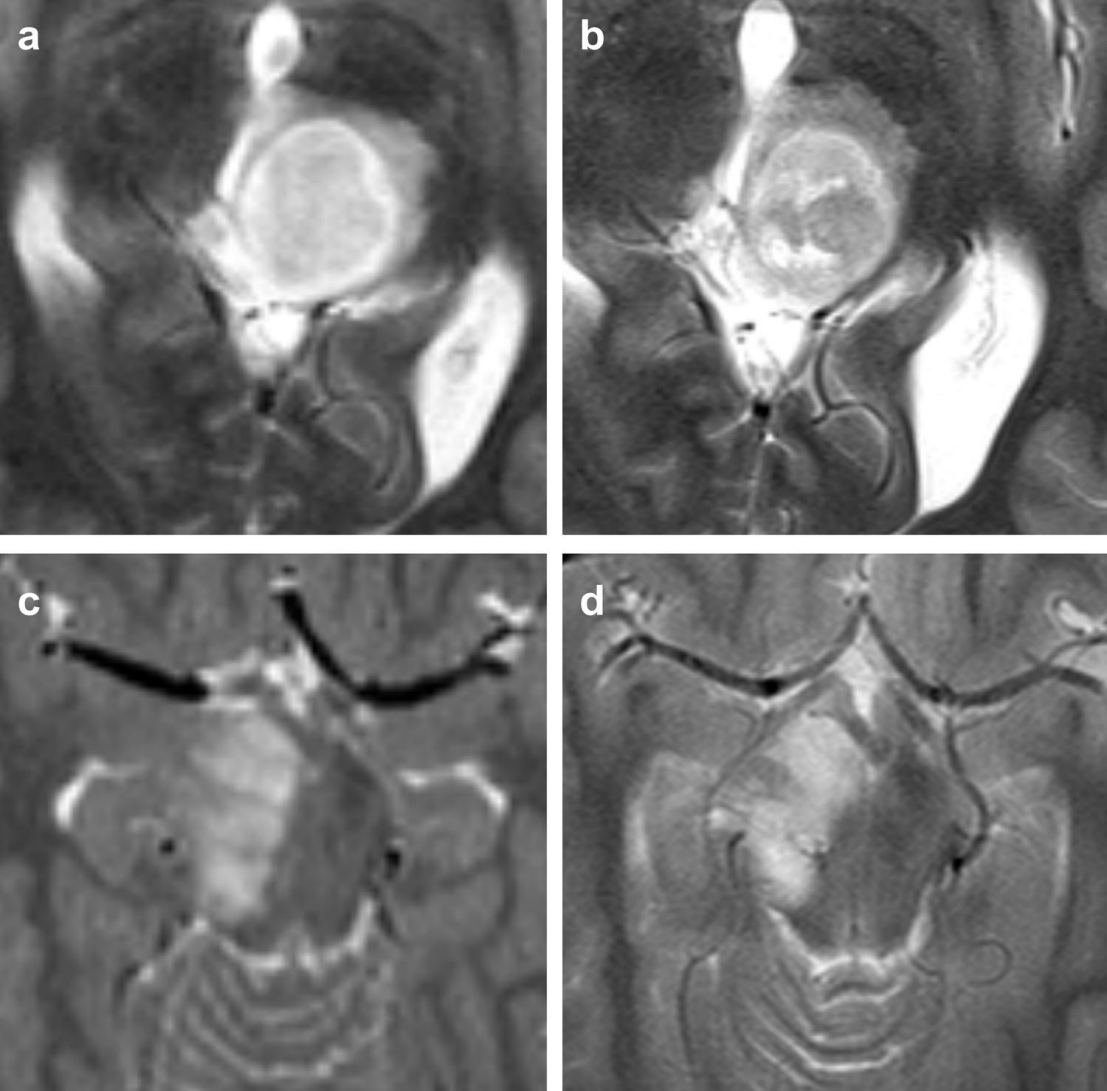
Two cases with stable disease post-radiotherapy. A 13-year-old female patient was diagnosed with biopsy-confirmed pilocytic astrocytoma. **a** Midline location indicated photon-based radiotherapy. **b** Twenty-four months after radiotherapy, no significant reduction in tumor volume was observed, though a slight reduction in T2 signal intensity (T2SI) was noted. **c** The second case presents a 9-year-old female patient with a low-grade glioma in the mesencephalon. Due to tumor progression, photon radiotherapy was initiated three years after diagnosis. **d** Eighteen months post-radiotherapy, both tumor volume and T2SI remained stable

**Fig. 6:**
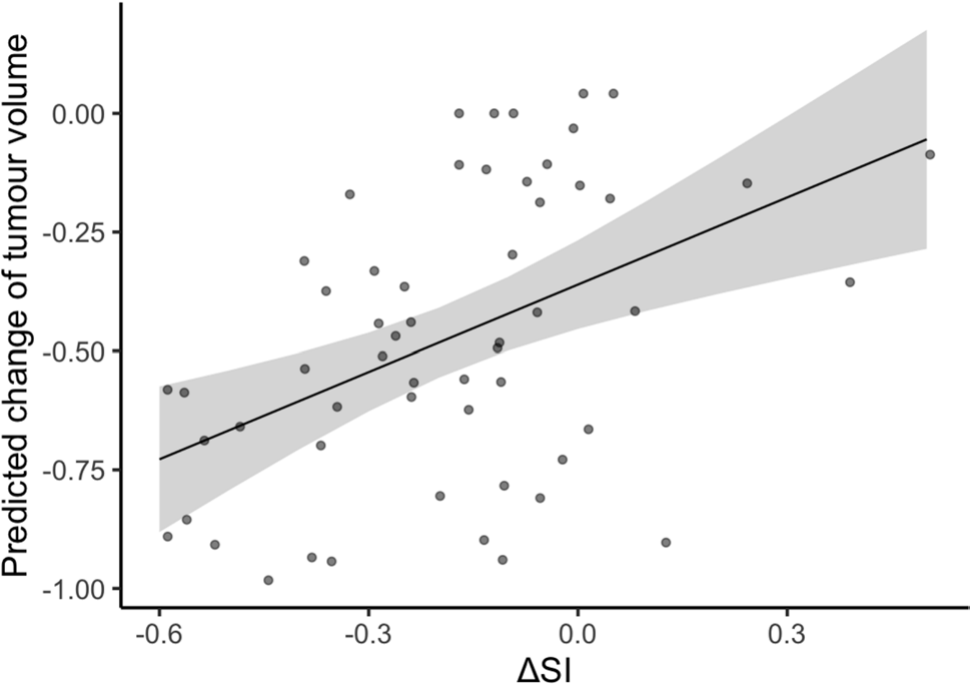
Relationship between T2-signal intensity reduction and tumor volume change. Effect plot showing the linear regression model between changes in T2-signal intensity (ΔSI) and tumor volume reduction. A significant positive association was observed (β = 0.61, *p* < 0.001), indicating that a decrease of 1.0 unit in SI corresponded to an average reduction of 0.61 units in tumor volume. The model explained approximately 20% of the variance in tumor volume change (adjusted R^2^ = 0.20), supporting SI reduction as an indicator of positive therapy response

ΔT2SI correlated with the extent of tumor volume decrease post-RT (r = 0.46, *p* < 0.001) and response assessment (ρ = 0.51; *p* < 0.001; Fig. 3). The decrease in T2SI was significantly pronounced for tumors that exhibited substantial volume reduction (MR, PR and CR; n = 40) compared to those with tumor stabilization (SD; n = 16; *p* < 0.001) post-RT. Cases with a representative course of T2SI are presented in Figs. 4 and 5. Reduction of T2SI predicted positive response to therapy in a simple linear regression analysis (dependent variable: tumor volume change; independent variable: ΔT2SI; β = 0.61, *p* < 0.001; adjusted R^2^ = 0.20; Table 3 and Fig. 6).

**Table 3:** Results of a simple linear regression model with the change of tumor volume as dependent variable and ΔT2SI as predictor

Coefficients	Estimate	Standard error	t-value	*p*-value
(intercept)	− 0.361	0.047	− 7.76	**< 0.001**
ΔT2SI	0.612	0.160	3.82	**< 0.001**

Residual standard error: 0.272 on 54 degrees of freedom, multiple R^2^: 0.213, adjusted R^2^: 0.198, F-statistic: 14.58 on 1 and 54 DF, *p*-value: < **0.001**;

*ΔT2SI* Difference of the signal intensity of the tumor in the last available dataset compared to baseline

The parameters sex (*p* = 0.37), tumor localization (*p* = 0.96), histology (*p* = 0.52), intratumoral susceptibility (*p* = 0.39), type of RT (*p* = 0.42), and pseudoprogression (*p* = 0.91) had no influence on ΔT2SI (Table 2).

Notably, the presence or absence of intratumoral susceptibility at baseline had no effect on the response to therapy (*p* = 0.71).

At the time pseudoprogression occured (n = 25), there was no significant change in T2SI (t6: n = 22 [88.0%]; t12: n = 3 [12.0%]) compared to baseline MRI (ΔT2SI: − 0.016 ± 0.261, *p* = 0.76). At pseudoprogression, 12/25 tumors showed enlarged T2-lesions (tumor volume and/or edema), while 13/25 patients showed enlarged contrast enhancing lesions or increased contrast uptake within the tumor. Both, an increase in T2-lesion and contrast enhancement was present in 1/25.

## Discussion

This is the first study to evaluate the correlation between T2SI tumor changes and positive responses, including cases of SD, to radiotherapy in pLGG. Based on our retrospective imaging analysis following front-line PBT and XRT, we demonstrate that the decrease in T2SI corresponds to the extent of post-treatment tumor size reduction.

Current guidelines recommend assessing pLGG size on T2WI [14]. While such measurements consider tumor dimensions, concomitant changes in tumor appearance on T2WI have not been systematically studied. Moreover, current neuroradiologic literature concentrates mainly on cohorts and advanced imaging techniques that aim almost exclusively to differentiate progression from pseudoprogression [21–23]. Studies addressing response to therapy by conventional MR-sequences in brain tumors are sparse. Still, the assessment of tumor characteristics based on conventional T2WI remains relevant in clinical routine. The visual (qualitative) assessment of T2WI is inherently subjective. Therefore, we implemented an easily available quantitative measurement in this work to reduce the impact of the rater assessment. Two independent readers performed quantitative assessment by placing representative ROIs.

At diagnosis, the imaging appearance of pLGG is well characterized; pLGG typically appear markedly hyperintense relative to cortex on T2WI [9, 10]. Our qualitative rating showed hyperintensity in nearly 90% of all pLGG types. Quantitatively, the tumor‑to‑cortex T2SI ratio at baseline was > 1 (mean 1.70 ± 0.44), consistent with hyperintensity. The agreement between the qualitative and quantitative assessment was excellent (AUC 0.91). The inter-rater reliability for the quantitative measurements of T2SI and ΔT2SI ranged between good and moderate (ICC 0.73–0.80) [20].

Following RT, the qualitative assessment of the tumors revealed a decrease of T2SI on the last available MRI within the 24 months observation period in slightly over half of the cohort (n = 30; 53.6%). Almost all cases achieved MR, PR and CR (29/30 cases). On quantitative analysis, a stronger decrease in T2SI was associated with a greater likelihood of significant tumor volume reduction. A recent study in diffuse intrinsic pontine glioma showed that combining T2SI measurements with tumor volume accurately predicts post‑RT response [24]. In that cohort, tumors with a response lower than PR (i.e., MR or SD) also exhibited significantly lower T2SI. Therefore, the T2SI reduction after RT is not only a phenomenon in pLGG.

A single patient of our cohort achieved CR with the highest decrease in T2SI at the last time-point with visible tumor tissue. In our study, ΔT2SI significantly correlated with the extent of tumor volume reduction. In contrast to the literature in brain tumors, a larger number of studies deal with conventional sequences in the field of body radiology. An MRI tumor regression grading system was introduced to assess therapy response after chemotherapy for anal canal carcinomas, correlating changes in T2-signal during therapy, in addition to size, with a positive treatment response [25]. This system was also applied to evaluate how much tumor mass transformed into regressive, and thus, T2-hypointense, portions [26]. In that cohort, cases with complete response showed approximately 50% signal decrease. Our cases with PR showed a decrease of − 28.3%. The observed signal decrease may potentially represent residual, but inactive tumor tissue. However, no histological evidence is available to confirm this, and it will likely remain difficult to substantiate in the future, as surgical intervention is not indicated in these cases.

Only minimal signal reduction was found in patients with SD, non-significant compared to their baseline T2SI. While not consistently scored as positive response to therapy in the literature, stable disease was considered as success of treatment in the HIT-LGG-1996-study [27], and this definition has been widely adopted for pLGG in the meantime [28, 29].

Interestingly, we observed the most pronounced decrease in T2SI at 18 and 24 months after RT, whereas no significant signal reduction was detected at 6 months post-RT. This suggests that RT exerts a long-term effect on tumor tissue.

In a single case, both raters qualitatively assessed an increase in T2SI during follow-up, whereas the corresponding quantitative measurement showed no significant change. The qualitative rating may have been confounded by pseudoprogression, which induced structural intratumoral changes at that time-point. This discrepancy suggests that quantitative, ROI‑based assessment is more robust, although it remains sensitive to regional tumor heterogeneity. In conclusion, congruent qualitative and quantitative T2SI assessments at baseline supports the validity of the approach.

Within our cohort, some patients developed transient post-therapeutic imaging changes that were classified as true pseudoprogression in our previous report [15]. At the time pseudoprogression was suspected, T2SI did not differ from baseline (no increase or decrease was observed), whereas contrast enhancement increased in 13/25 tumors. Thus, our data complement the established findings that increasing contrast enhancement in LGG post-RT is not an indicator for progression [13, 30]. Within the true pseudoprogression group, tumors achieving an unequivocal volume reduction in their latest MRI also showed a significant decrease in T2SI.

Although one might expect tumors with susceptibility foci or lower T2SI at baseline to regress less, our data do not support this hypothesis. Tumor volume reduction did not correlate with baseline T2SI. Even the uncommon subset of pLGGs that were T2-isointense to cortex—expected to represent more inactive tissue—showed volume decreases comparable to their hyperintense counterparts. Likewise, the presence of intratumoral susceptibility, often a marker of calcification and presumed inactivity in LGG [31], had neither effect on subsequent ΔT2SI nor on response to therapy.

Our conclusions are limited by the retrospective study design as well as by the lack of complete imaging series within the 24 months follow-up period. Further, the multicenter approach of our study was accompanied by differences of scanners in use, magnetic field strengths, and sequence parameters which could have introduced a quantitative bias, because different imaging techniques may result in different tissue contrasts. The lack of standardization in imaging parameters likely impeded the application of a uniform ROI-based analysis or automated quantification. Additionally, although a decrease in T2SI suggests regressive tumor tissue, histological confirmation is lacking.

Since qualitative changes of T2SI are easily assessable, implementation of their use in clinical routine may be facilitated. In view of the simplicity of the approach, this work may impact upon patient care by supporting imaging evaluation especially in centers with limited scanning capacities. Furthermore, automatic tissue segmentation, rather than manual ROI placement, should objectify results in the future and minimize rater bias.

## Conclusions

This study demonstrates that a reduction in T2SI on conventional T2-weighted MRI correlates with a significant decrease in tumor volume following RT in pLGG. In cases of post-radiotherapy pseudoprogression, relative T2SI remains stable despite increased contrast enhancement or apparent tumor enlargement.

By quantitatively assessing T2SI through standardized ROI placement in a multireader setting, we provide preliminary evidence that T2SI may serve as a complementary imaging biomarker alongside conventional volumetric analysis, potentially enhancing diagnostic confidence especially in case of pseudoprogression. Further studies are warranted to evaluate the prognostic utility of T2SI in distinguishing true tumor progression from pseudoprogression in pLGG.

## Appendix

See Fig. 6

See Table 3

## References

[1] Ostrom QT, de Blank PM, Kruchko C et al (2015) Alex’s lemonade stand foundation infant and childhood primary brain and central nervous system tumors diagnosed in the United States in 2007–2011. Neuro Oncol 16(Suppl):x1–x36. 10.1093/neuonc/nou327 25542864 10.1093/neuonc/nou327 PMC4277295

[2] Sievert AJ, Fisher MJ (2009) Pediatric low-grade gliomas. J Child Neurol 24:1397–1408. 10.1177/0883073809342005 19841428 10.1177/0883073809342005 PMC2917804

[3] de Blank P, Bandopadhayay P, Haas-Kogan D et al (2019) Management of pediatric low-grade glioma. Curr Opin Pediatr 31:21–27. 10.1097/mop.0000000000000717 30531227 10.1097/MOP.0000000000000717 PMC6664811

[4] Boop S, Shimony N, Boop F (2024) How modern treatments have modified the role of surgery in pediatric low-grade glioma. Childs Nerv Syst. 10.1007/s00381-024-06412-w 38676718 10.1007/s00381-024-06412-w PMC11511694

[5] Merchant TE, Conklin HM, Wu S et al (2009) Late effects of conformal radiation therapy for pediatric patients with low-grade glioma: prospective evaluation of cognitive, endocrine, and hearing deficits. J Clin Oncol 27:3691–3697. 10.1200/JCO.2008.21.2738 19581535 10.1200/JCO.2008.21.2738 PMC2799064

[6] Williams NL, Rotondo RL, Bradley JA et al (2018) Late effects after radiotherapy for childhood low-grade glioma. Am J Clin Oncol 41:307–312. 10.1097/COC.0000000000000267 26808258 10.1097/COC.0000000000000267

[7] Saad S, Wang TJC (2015) Neurocognitive deficits after radiation therapy for brain malignancies. Am J Clin Oncol 38:634–640. 10.1097/COC.0000000000000158 25503433 10.1097/COC.0000000000000158

[8] Grabenbauer GG, Schuchardt U, Buchfelder M et al (2000) Radiation therapy of optico-hypothalamic gliomas (OHG)–radiographic response, vision and late toxicity. Radiother Oncol 54:239–245. 10.1016/s0167-8140(00)00149-3 10738082 10.1016/s0167-8140(00)00149-3

[9] Chen J, Qi X, Zhang M et al (2023) Review on neuroimaging in pediatric-type diffuse low-grade gliomas. Front Pediatr 11:1149646. 10.3389/fped.2023.1149646 37920791 10.3389/fped.2023.1149646 PMC10619148

[10] Opancina V, Esposito S, Di Meco F et al (2023) Magnetic resonance imaging characteristics of pediatric pilocytic astrocytoma. Neurol Sci 44:4033–4040. 10.1007/s10072-023-06893-8 37322312 10.1007/s10072-023-06893-8

[11] Su Y, Kang J, Lin X et al (2023) Whole-tumor histogram analysis of diffusion and perfusion metrics for noninvasive pediatric glioma grading. Neuroradiology 65:1063–1071. 10.1007/s00234-023-03145-6 37010573 10.1007/s00234-023-03145-6

[12] Coakley KJ, Huston J III, Scheithauer BW et al (1995) Pilocytic astrocytomas: well-demarcated magnetic resonance appearance despite frequent infiltration histologically. Mayo Clin Proc 70:747–751. 10.4065/70.8.747 7630212 10.4065/70.8.747

[13] Gaudino S, Quaglio F, Schiarelli C et al (2012) Spontaneous modifications of contrast enhancement in childhood non-cerebellar pilocytic astrocytomas. Neuroradiology 54:989–995. 10.1007/s00234-012-1010-3 22286205 10.1007/s00234-012-1010-3

[14] Fangusaro J, Witt O, Hernáiz Driever P et al (2020) Response assessment in paediatric low-grade glioma: recommendations from the response assessment in Pediatric Neuro-Oncology (RAPNO) working group. Lancet Oncol 21:e305–e316. 10.1016/S1470-2045(20)30064-4 32502457 10.1016/S1470-2045(20)30064-4

[15] Stock A, Hancken C-V, Kandels D et al (2022) Pseudoprogression is frequent after front-line radiation therapy in pediatric low-grade glioma: results from the German Low-Grade Glioma Cohort. Int J Radiat Oncol Biol Phys 112:1190–1202. 10.1016/j.ijrobp.2021.12.007 34933039 10.1016/j.ijrobp.2021.12.007

[16] Ludmir EB, Mahajan A, Paulino AC et al (2019) Increased risk of pseudoprogression among pediatric low-grade glioma patients treated with proton versus photon radiotherapy. Neuro Oncol 21:686–695. 10.1093/neuonc/noz042 30753704 10.1093/neuonc/noz042 PMC6502497

[17] Tsang DS, Murphy ES, Lucas JT Jr et al (2017) Pseudoprogression in pediatric low-grade glioma after irradiation. J Neurooncol 135:371–379. 10.1007/s11060-017-2583-9 28752498 10.1007/s11060-017-2583-9 PMC8717050

[18] Naftel RP, Pollack IF, Zuccoli G et al (2015) Pseudoprogression of low-grade gliomas after radiotherapy: pseudoprogression after radiotherapy. Pediatr Blood Cancer 62:35–39. 10.1002/pbc.25179 25213668 10.1002/pbc.25179

[19] Kandels D, Pietsch T, Bison B et al (2020) Loss of efficacy of subsequent nonsurgical therapy after primary treatment failure in pediatric low-grade glioma patients-report from the German SIOP-LGG 2004 cohort. Int J Cancer 147:3471–3489. 10.1002/ijc.33170 32580249 10.1002/ijc.33170

[20] Koo TK, Li MY (2016) A guideline of selecting and reporting intraclass correlation coefficients for reliability research. J Chiropr Med 15:155–163. 10.1016/j.jcm.2016.02.012 27330520 10.1016/j.jcm.2016.02.012 PMC4913118

[21] Hilario A, Salvador E, Cardenas A et al (2024) Low rCBV values in glioblastoma tumor progression under chemoradiotherapy. Neuroradiology 66:317–323. 10.1007/s00234-023-03279-7 38183424 10.1007/s00234-023-03279-7

[22] Stadlbauer A, Eyüpoglu I, Buchfelder M et al (2019) Vascular architecture mapping for early detection of glioblastoma recurrence. Neurosurg Focus 47:E14. 10.3171/2019.9.focus19613 31786560 10.3171/2019.9.FOCUS19613

[23] van West SE, de Bruin HG, van de Langerijt B et al (2017) Incidence of pseudoprogression in low-grade gliomas treated with radiotherapy. Neuro Oncol 19:719–725. 10.1093/neuonc/now194 28453748 10.1093/neuonc/now194 PMC5464441

[24] Yu X, Li S, Mai W et al (2024) Pediatric diffuse intrinsic pontine glioma radiotherapy response prediction: MRI morphology and T2 intensity-based quantitative analyses. Eur Radiol 34:7962–7972. 10.1007/s00330-024-10855-9 38907098 10.1007/s00330-024-10855-9 PMC11557687

[25] Kochhar R, Renehan AG, Mullan D et al (2017) The assessment of local response using magnetic resonance imaging at 3- and 6-month post chemoradiotherapy in patients with anal cancer. Eur Radiol 27:607–617. 10.1007/s00330-016-4337-z 27090113 10.1007/s00330-016-4337-z PMC5209434

[26] Kluza E, Rozeboom ED, Maas M et al (2013) T2 weighted signal intensity evolution may predict pathological complete response after treatment for rectal cancer. Eur Radiol 23:253–261. 10.1007/s00330-012-2578-z 22777621 10.1007/s00330-012-2578-z

[27] Gnekow AK, Falkenstein F, von Hornstein S et al (2012) Long-term follow-up of the multicenter, multidisciplinary treatment study HIT-LGG-1996 for low-grade glioma in children and adolescents of the German Speaking Society of Pediatric Oncology and Hematology. Neuro Oncol 14:1265–1284. 10.1093/neuonc/nos202 22942186 10.1093/neuonc/nos202 PMC3452343

[28] Hessissen L, Parkes J, Amayiri N et al (2017) SIOP PODC adapted treatment guidelines for low grade gliomas in low and middle income settings. Pediatr Blood Cancer 64(Suppl 5):e26737. 10.1002/pbc.26737 10.1002/pbc.26737 29297618

[29] Kelety T, Thomale U-W, Kandels D et al (2024) Adaption of neurosurgical resection patterns for pediatric low-grade glioma spanning two decades-report from the German LGG-studies 1996–2018. Cancer Med 13:e7417. 10.1002/cam4.7417 38923198 10.1002/cam4.7417 PMC11194681

[30] Bison B, Warmuth-Metz M, Schneckenburger M et al (2011) Contrast enhancement of low grade gliomas (LGG) during follow-up. Neuroradiol J 24:415–418. 10.1177/197140091102400310 24059664 10.1177/197140091102400310

[31] Lee YY, Van Tassel P, Bruner JM et al (1989) Juvenile pilocytic astrocytomas: CT and MR characteristics. AJR Am J Roentgenol 152:1263–1270. 10.2214/ajr.152.6.1263 2718863 10.2214/ajr.152.6.1263

